# Characterization of contact resistances in ceramic-coated vertically aligned carbon nanotube arrays†

**DOI:** 10.1039/c8ra10519g

**Published:** 2019-03-04

**Authors:** Meng Li, Ning Yang, Vanessa Wood, Hyung Gyu Park

**Affiliations:** Department of Mechanical and Process Engineering, ETH Zürich Zürich CH-8092 Switzerland parkh@ethz.ch; Department of Information Technology and Electrical Engineering, ETH Zürich Zürich CH-8092 Switzerland vwood@ethz.ch

## Abstract

Despite the technological significance of carbon nanotube (CNT) arrays and metal-oxide coated CNTs for electronic and electrochemical devices such as supercapacitors, lithium-ion batteries, and solar-chemical cells, sub-optimal device performance often results due to large contact resistance between the CNTs and the metallic current collectors or between the CNTs and their ceramic coatings. While contact resistance measurements are regularly carried out on individually contacted CNTs, contact resistance measurements on vertically aligned (VA) CNT arrays are not routine. Here, we demonstrate that two-probe electrical current–voltage measurements and electrochemical impedance spectroscopy can be used to probe the end contact resistance and side contact resistances of coated and uncoated VACNT arrays in order to optimize material deposition and selection.

## Abstract 2

End- and side-contact resistances between carbon nanotubes and contacts are easily probed in vertically aligned arrays.

## Graphical abstract

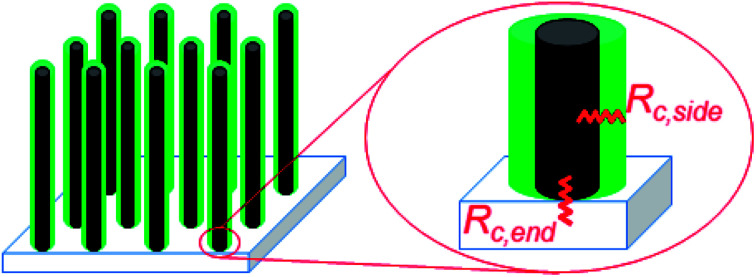

## 1. Introduction

With highly directional transport and a large graphitic surface area for functionalization, vertically aligned carbon nanotube (CNT) arrays have outstanding electrical and physical properties^1–3^ for a number of different types of devices.^4^ For instance, it has been predicted that a densely packed forest of vertically aligned CNTs (VACNTs) could outperform Cu as microelectronic interconnects.^5^ CNTs decorated with conductive polymers or inorganic oxides have applications in electrochemical devices such as supercapacitors,^6,7^ dye-sensitized solar cells,^8^ and water splitting cells.^9^ Used as a conductive additive, CNTs help lower electrode resistance to enhance capacitive deionization performance for salt water desalination,^10^ or increase the rate performance and cyclability in lithium-ion batteries.^11^ Given the low bulk resistance of the CNTs, the contact resistance they form at the interface with charge collectors or surface coatings play a critical role in the overall device performance.

In order to minimize ohmic losses, it is necessary for the CNTs to be electrically connected with minimal resistance to the charge collectors and for coatings to be electrically connected with minimal resistance to the CNTs. Although a theoretical quantum contact resistance of a single-walled carbon nanotube (SWCNT) is ∼6.45 kΩ,^12^ experimentally measured contact resistances span many orders of magnitude.^13,14^ For multi-walled CNTs (MWCNTs), contact resistance variations can be even greater. For example, a MWCNT (∼100 nm in diameter) semi-spherically coated with Ti/Au over a large contact area, contact resistance (*R*_c_) has been reported to be 1.56 kΩ.^15^ However, a resistance value of ∼1 GΩ has been reported for the case where the tube is placed directly on small, pre-deposited Au finger electrodes without any further treatment.^16^ Reasons for these large discrepancies (in addition to differences in the CNTs themselves) include differences in contact area and configuration (side- or end contact), physio-chemical parameters of the contact (*e.g.*, work function and wettability),^17^ and the type of interfacial contact (*e.g.*, quantum mechanical tunneling^18–20^*vs.* classical Schottky junction^21,22^).

Most reports^13–16,23–30^ measure the *R*_c_ of a suspended CNT, which involves use of advanced nanofabrication and characterization such as *in situ* transmission electron microscopy (TEM) or conductive atomic force microscopy (AFM). Although quite precise, these methods are usually costly and time-consuming. On a device scale, the ensemble-averaged, end-contact resistance per nanotube can be obtained from a polished VACNT forest less than a micrometer in height.^17,31,32^ This approach loses accuracy the taller the CNT forest (tall CNT forests offer higher mass loading in electrochemical devices^33^) because their lengths become the more heterogeneous.^34,35^

**Fig. 1:**
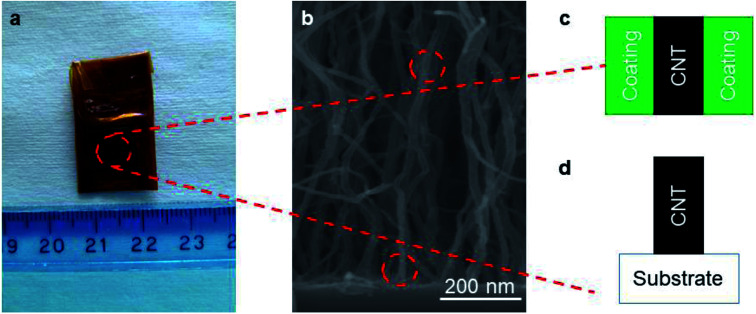
(a) Photograph of a typical TiO_2_/VACNT electrode, (b) SEM image of TiO_2_-coated VACNTs. Schematics of (c) side contact between CNT side wall and oxide coating and (d) end contact between CNT bottom end and current collector.

Here, we establish characterization methods for both side- and end-contact resistances of VACNT forests (Fig. 1). We show that two-probe electrical measurements can be used to determine contact resistances between the end of the CNTs and the current collector. It is further possible to determine the effective spacing between the metallic current collector and the CNTs, which relates to the electronic structure of the substrate and the wetting of the metal and the CNTs. To determine side contact resistances between a coating and a CNT sample, we show that electrochemical techniques (cyclic voltammetry and electrochemical impedance spectroscopy) enable the decoupling of impedance contributions from different origins. Combined with knowledge of the average length and number of CNTs in the array, the resistivity of the coating itself can also be determined.

## 2. Experimental

### 2.1 Material synthesis

To grow a VACNT array, a catalyst layer consisting of a (nominal) 3 nm-thick Fe layer atop a 20 nm-thick Al barrier film was deposited *via* e-beam evaporation on Si chips (0.5 mm in thickness) or on Ni foils (Alfa Aesar, 99% metal basis, 0.025 mm in thickness). Prior to the catalyst deposition, the Ni foil surface was physically etched away with mild Ar^+^ beam milling in order to remove contaminants. The catalyst samples were loaded into a quartz tube furnace, heated up to 750 °C at 30 °C min^−1^ at ambient pressure with a flow of 600 sccm of H_2_ and 400 sccm of Ar, and annealing for 20 min at these conditions. After annealing, C_2_H_4_ (250 sccm) was added to the gas ambient to grow VACNT forests. TiO_2_ was coated on the as-grown VACNTs *via* plasma-enhanced atomic layer deposition (ALD) at 120 °C in an Oxford Instrument AL1 FlexAL system. Each cycle consisted of a 1.5 s dose (at 200 sccm) of Tetrakis(dimethylamino)titanium as a Ti precursor and a 10 s dose (at 20 sccm) of ozone plasma as an oxygen source.

### 2.2 VACNT transplantation

VACNT transplantation was done *via* a poly(methyl methacrylate) (PMMA) assisted stamping procedure described previously.^8^ The as-grown CNT samples were pressed into a coating (∼1 μm in thickness) of PMMA on different conductive substrates, *i.e.* FTO glass, W coated glass or Ni foil, before curing and peeling off. The PMMA layer was then pyrolized by annealing in 900 sccm H_2_/100 sccm Ar at 400 °C for 2 h. The pyrolysis process removes most of the PMMA,^36^ and thus does not interfere with the electrical or weight measurements. The transferred CNTs were weighed on a new substrate with a Mettler Toledo XP2U ultra-micro balance (1 μg in resolution) before and after transplantation.

### 2.3 Characterizations

The height and alignment of the VACNTs were characterized using scanning electron microscopes (SEM, FEI Nova 450 and Hitachi SU 8200). TEM was used to obtain CNT diameter statistics (Philips CM12). Catalyst particle density was probed with a Bruker Fastscan AFM. The as-grown CNT quality and the phase information of the as-deposited TiO_2_ layer were determined by use of a Renishaw InVia Raman spectroscope (785 nm excitation) and NTMDT NTEGRA Raman spectroscope (571 nm excitation).

## 3. Results and discussion

### 3.1 VACNTs characterization

To perform macroscopic measurement on a VACNT array and then extract information about the average properties of individual tubes, we first need to know the properties of the CNTs, including their areal number density (*n*_CNT_), length (*l*), and linear resistivity (*ρ*′′). Literature has shown that the linear resistivity relies heavily on the CNT quality, such as wall number (*n*), diameter and defect density,^37,38^ and is usually described by a charge mean-free-path theory.^14,39^

**Fig. 2:**
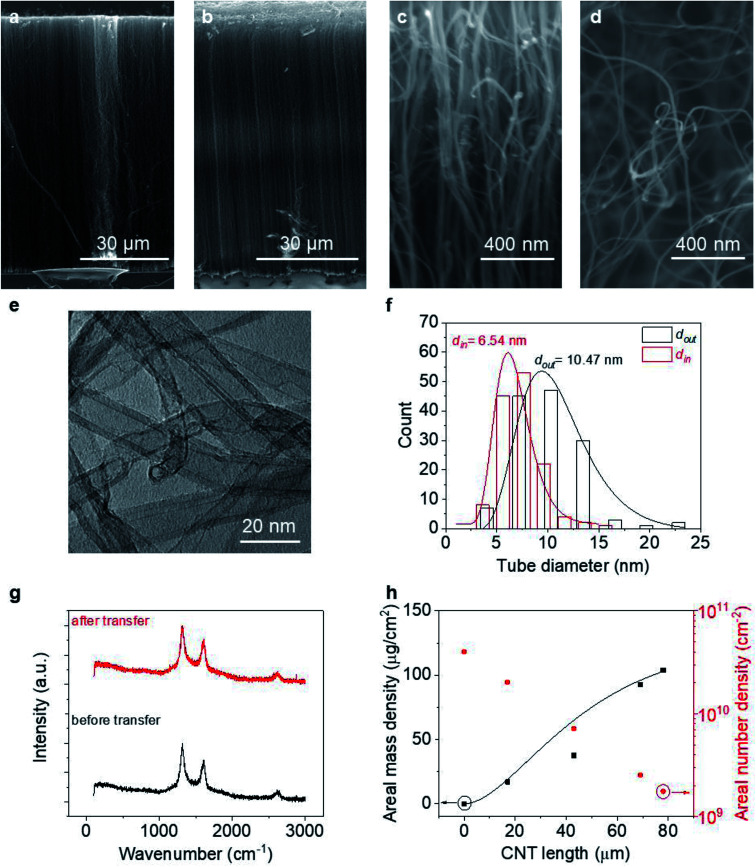
CNT characteristics: SEM images of the VACNT forest before (a) and after transplantation (b). High magnification images near the top (c) and bottom (d) of the forest, prior to transplantation; (e) typical TEM image of CNTs (f) inner and outer diameter statistics; (g) Raman spectra of CNTs before and after transfer. (h) Areal mass and number density of CNTs in VACNT forest.

Fig. 2 provides information about the VACNT forests investigated in this work. As can be seen from Fig. 2c and d, the VACNTs near the top of the forest are more aligned, while they are less dense and more entangled near the bottom, consistent with a density decay regime.^40,41^ After transplantation (where the “top” and “bottom” end of the CNTs are reversed), both the forest height (*L*) and alignment are well preserved with minimal bending at the top likely caused by decreased CNT number density or small pressures applied during the transfer process. Statistics over 300 tubes grown in different batches reveal that the MWCNTs possess an average outer diameter (*d*_out_) of 10.2 ± 0.42 nm and inner diameter (*d*_in_) of 6.0 ± 0.47 nm (more details can be found in Fig. S1†). With ∼0.34 nm as the interlayer spacing between CNT walls,^42^ an averaged *n* of each CNT is estimated as ∼6. Raman spectra at the top and bottom of a CNT forest are similar with unchanged Raman D-to-G intensity ratio (∼1.35) along the thickness, suggesting an invariant defect density as well as *ρ*′′ along the CNTs.^37^ We note that the bottom end switches with the top after transplantation. Regardless of the growth time (*t*) or forest height, CNTs remain of similar characteristics in terms of tube diameter and quality (information about VACNTs of other thicknesses can be found in Fig. S1†).

Counting the number of CNTs from cross-sectional SEM images alone may not provide an accurate effective *n*_CNT_ because an SEM of a porous structure like a CNT forest contains depth information. A more accurate and reliable way is the weight-gain method,^43^ which allows us to describe *n*_CNT_ and model its dependency on *L*. Assuming the tube growth is self-terminated^44^ with a catalyst deactivation probability constant *a*, one can relate areal density *n*_CNT_ to growth time *t*:1
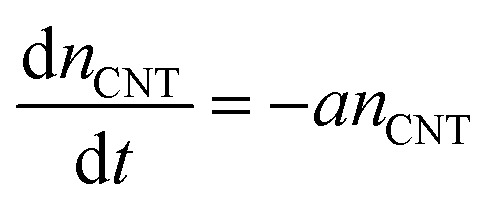


Neglecting the initial CNT self-organization period, *n*_CNT_ roughly equals to the catalyst number density (*N*_0_ = 4 × 10^10^ cm^−2^ from AFM data, Fig. S2†) at *t* = 0. Integration over eqn (1) gives:

2 *n*_CNT_ = *N*_0_ exp(−*at*)

The mass of the entire VACNT forest (*M*) over area (*A*) can then be written as the summation of all the nanotubes grown until *t*:3
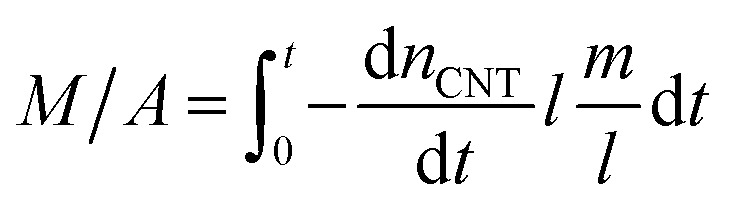


In this equation, *m* (in g) and *l* (in nm) are the mass and length of an individual tube at *t*. To proceed, two maneuvers can be applied. One is to set *l* = *vt*, where *v* is the steady-state growth rate of the VACNT forest (10 μm min^−1^ in this study). Another is to consider the specific surface area of a MWCNT referring to the literature^43^ (eqn (4)), where *D* is the aggregate diameter of all the carbon walls summed up (53 ± 2.5 nm from our TEM analysis):4
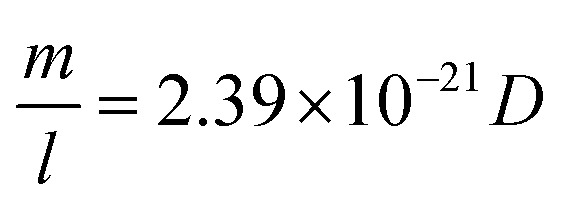


Combining eqn (1)–(4) and replacing the final *t* with *L*/*v*, we finally reach:5
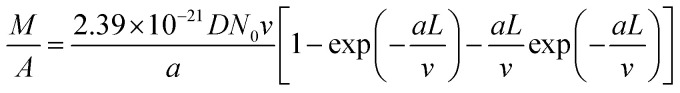


Fitting Fig. 2h with eqn (5) (black line) yields *a* = 0.40 min^−1^. Plugging this value to eqn (2), we can see that *n*_CNT_ drops by a factor of ∼20 when *L* becomes longer than ∼80 μm. The relatively short length (submillimeter) and low density (*O*(≤10^10^) cm^−2^) agree with previous findings in the literature that the growth of the VACNT forest is limited by catalyst poisoning.^45^ Extrapolation of eqn (5) predicts saturation of the CNT forest growth if *L* exceeds 150 μm (*M*/*A* reaching a plateau, and entering the termination stage^40^), which is in good agreement with our experimental data.

### 3.2 Measuring end contact resistance of VACNTs

**Fig. 3:**
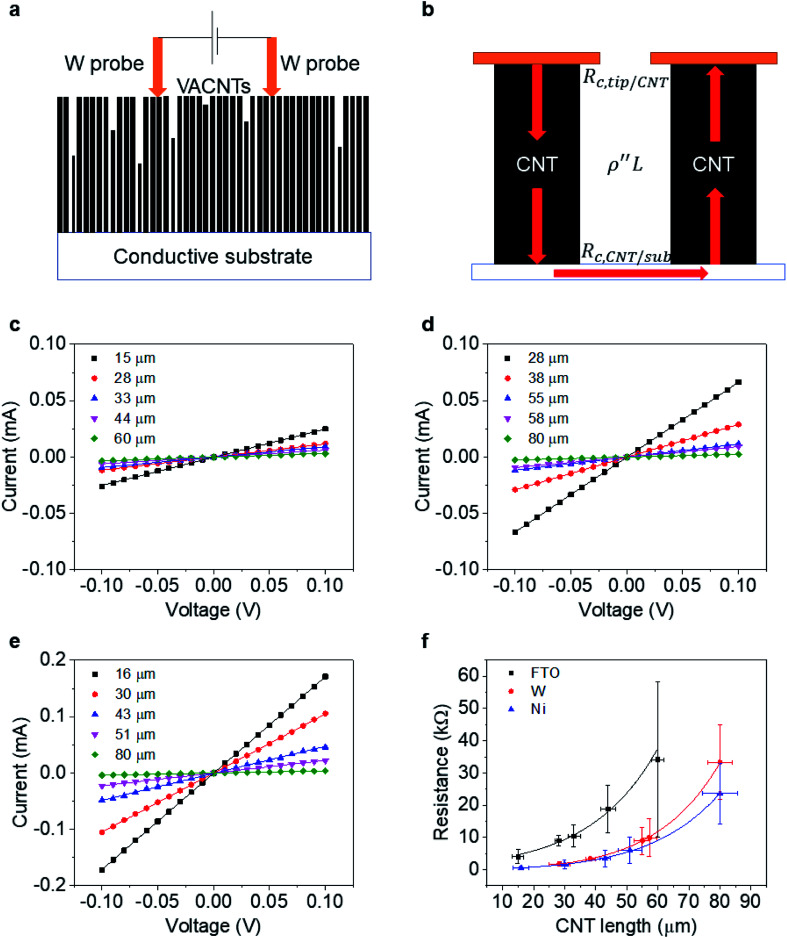
Measurements of VACNT end contact resistance: schematics (a) of the *I*–*V* test setup and (b) measurement with red arrows marking the electron flow; typical *I*–*V* curves of as-transplanted VACNTs in different length on (c) FTO, (d) W and (e) Ni substrates; and (f) plot of resistance *versus* CNT length for CNTs on FTO, W, and Ni.

To determine the end contact resistance, we use the two-probe electrical current–voltage measurements shown in Fig. 3a. The measurements are carried out with a probe station (Cascade) connected to a semiconductor device analyzer (Agilent B1500A). A pair of W probes (19 μm in diameter, giving *A*_probe_ = 2.83 × 10^−6^ cm^2^) is aligned horizontally with a separation distance of ∼100–200 μm and then carefully placed atop the CNT forest (approaching in 2 μm steps) to make direct electrical contact. Measurements are repeated at a minimum of 6 distinct positions on each sample.

When using this setup, electrons are injected from one W probe into multiple CNTs (about ∼ 10^4^) in parallel. CNTs much shorter than the average are not probed. As reported previously,^32^ the lateral resistance between CNTs in the VACNT forest is large enough to neglect. Most injected electrons thus travel across the bottom conductive substrate and exit from CNTs that contact another probe (Fig. 3b). Therefore, one can write the resistance (*R*) per individual CNT as:

6 *R* = *A*_probe_*n*_CNT_*R*_tot_/2 = *R*_c,tip-CNT_ + *ρ*′′*L* + *R*_c,CNT-sub_

*R*_c,tip-CNT_ and *R*_c,CNT-sub_ represent the contact resistances between the W tip and a CNT and between a CNT and the bottom substrate, respectively. The first term ideally remains a constant, and the latter depends solely on the nature of the interface between the CNT end and substrate. The sum of these two terms renders total contact resistance, *R*_c_.

Fig. 3c–e shows the *I*–*V* curves of as-transplanted VACNTs on various substrates. For any given VACNT height and bottom contact, a linear *I*–*V* curve is observed so *R*_tot_ is the inverse slope. Plotting *R*_tot_*vs.* the VACNT height shows a non-linear behavior (Fig. 3f). As discussed previously, while CNT quality (mainly *ρ*′′ and *n*) does not vary over *l* or *L*, *n*_CNT_ changes significantly. Therefore, to extract the contact resistance *R*_c_, we combine eqn (2) and (6):7
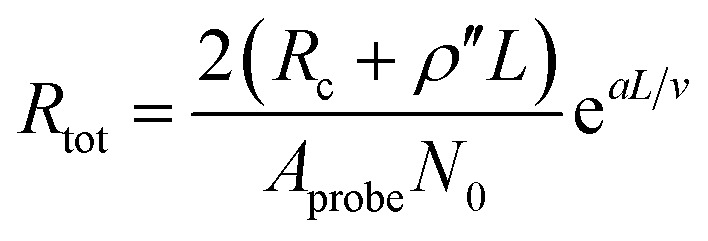
and fit this to the data in Fig. 3f.

The fitting results (Table 1) indicate a *ρ*′′ (∼1.15 MΩ μm^−1^) and *a* that are invariant with substrate material. This highlights the reliability of the fit since *ρ*′′ and *a* should indeed be intrinsic properties of the CNTs. The fitted values of *a* match reasonably well with the one from our modified weight-gain approach. At a small electric field, it is very unlikely that optical phonons and zone-boundary phonons play any significant role in electron transport,^39^ and therefore *ρ*′′ is dominated by acoustic phonons from scattering at the defects. The *ρ*′′ value of our MWCNTs is higher than the values measured by dipping a freestanding nanotube into liquid metals^2,25,48^ (200 Ω μm^−1^) or in a FET configuration (41 kΩ μm^−1^),^49^ but it is quite close to the values measured where the tube is curved by an AFM tip (1.93 MΩ μm^−1^)^50^ or under direct probing (1.39 MΩ μm^−1^).^51^ This finding suggests that in addition to the CNT quality, *ρ*′′ might also be sensitive to slight bending (Fig. 2b). In fact, theoretical modelling has shown that the resistance of CNT under mechanical deformation can change.^52^

**Table 1:** Fitting result of Fig. 3f. Fermi energies of FTO, W and Ni are from literature^46,47^

Substrate material	*E*_F_ (eV)	*R*_c_ (MΩ)	*ρ*′′ (MΩ μm^−1^)	*a* (min^−1^)	Gap distance (Å)
FTO	5.0	125.2	1.2	0.4	5.9
W	4.55	1.28	1.14	0.34	3.7
Ni	5.15	0.4	1.11	0.38	—

However, *R*_c_ varies by orders of magnitudes for the different substrates (Ni, W, and FTO). On Ni, the contact resistance is the lowest with *R*_c_ = 0.4 MΩ. Metals with unfilled d-orbitals (Ni has electron configuration 3d^8^4s^2^) have a strong affinity to carbon atoms^53^ and can potentially form carbides (covalent bonds) upon annealing. Experiments^29,54^ have shown that such carbide formation is particularly helpful to obtain a low resistance ohmic contact. Since it is the theoretical quantum resistance for a SWCNT is 6.45 kΩ (ref. 12) and multiple conduction pathways (∼2 for our MWCNT), we expect Ni-MWCNT contact, *R*_c,CNT-sub_, to small enough to be omitted. We can assign the 0.4 MΩ to the contact resistance between the W probes and the VACNT forest (*R*_c,tip-CNT_). This allows us to decouple the *R*_c,CNT-sub_ from *R*_c,tip-CNT_ for the case of W or FTO substrates.

The value of *R*_c,CNT-sub_ for FTO (125.2–0.4 = 124.8 MΩ) is larger than that for W (1.28–0.4 = 0.88 MΩ), which can be attributable to a lack of d vacancies in FTO^53^ and the relatively weaker carbon affinity to 5d vacancies in W.^17^ In such a case, the weak bonding is partially van der Waals in nature and can be thought of as an average bonding length (or vacuum gap) of *s* (in Å) through which electron tunneling occurs.^19^ At small electrical bias (in our case *V* ≤ 0.1 V), Simmons^55^ has derived eqn (8) for electron tunneling between two dissimilar electrodes, in which *φ* (in eV) is the averaged work function, and *J* the current density (in A cm^−2^):8
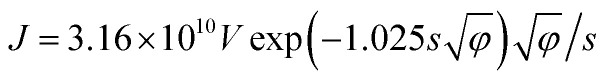
Here, *J* is linearly proportional to *V*. Given the end-contact area of an individual CNT (*A*_CNT_ ∼ 5 × 10^−13^ cm^2^), one can rearrange eqn (8) into (9):9
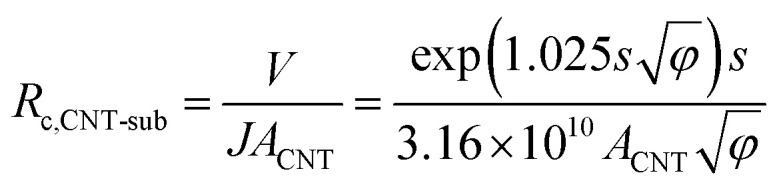


Since both *φ* and *s* appear in the exponent, the resistance can differ substantially by the choice of metal leads, consistent with our findings (Table 1). Fitting by eqn (9) gives a tunneling gap of ∼3.7 Å in W and ∼5.9 Å in FTO. In Fig. S3,† we see that as-transplanted CNTs on the FTO substrate detach easily after soaking into water, in contrast to their stability on Ni. This observation supports the fact that CNT bond less strongly to FTO. In short, in order to minimize *R*_c_, it is crucial to have good wetting and to shorten the tunneling gap spacing with metals such as Ti,^15,26,56,57^ Ni,^49,58^ and Pd.^30^

### 3.3 Measuring side contact resistance of coated VACNTs

**Fig. 4:**
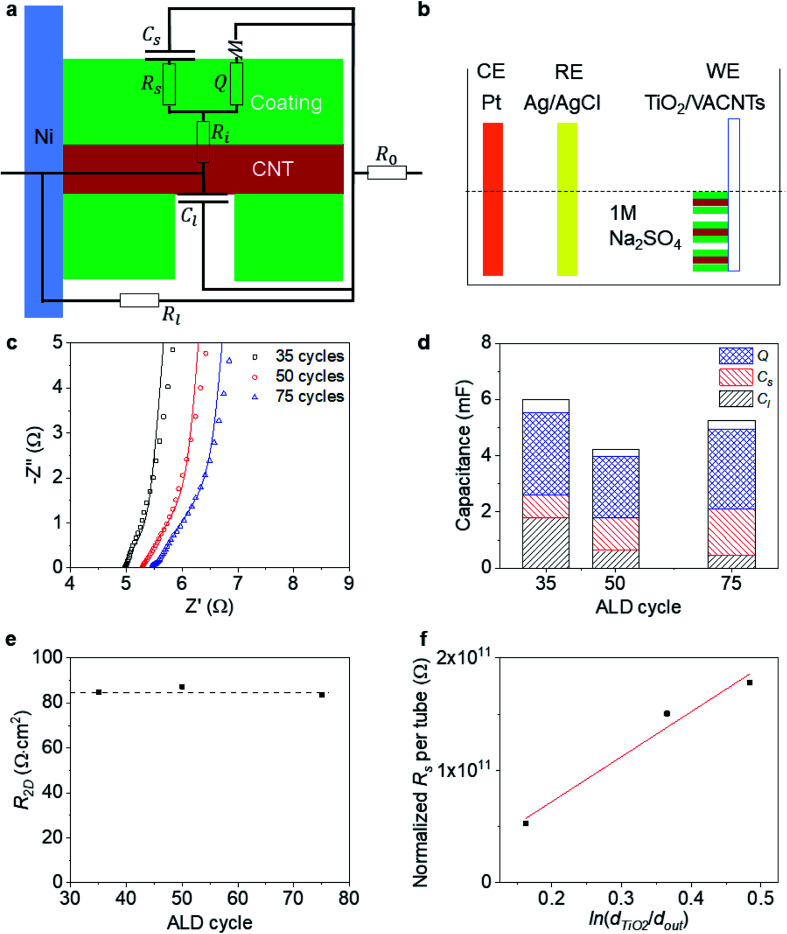
Measurements of VACNT side contact resistance: (a) schematic of a partially TiO_2_-coated CNT and its equivalent circuit diagram; (b) schematic of the EIS test setup; (c) Nyquist spectra of TiO_2_/VACNT samples; (d) bar plot of capacitance contributions of TiO_2_/VACNT samples, with the total capacitance from CV shown with the blank bar; (e) 2D resistance at the interface of TiO_2_ and CNT for numbers of ALD cycles; and (f) normalized electrical resistance of TiO_2_ per tube with respect to coating thickness and tube diameter.

To probe the side-contact resistance between a CNT and a coating, we propose electrochemical characterization. We are faced with the geometry shown in Fig. 4a and wish to extract the interface resistance *R*_i_ and normalize it with the areal coverage of the coating *A*_coat_ to obtain the 2D resistance *R*_2D_. To do so, we use electrochemical impedance spectroscopy (EIS) and cyclic voltammetry (CV). By performing these measurements on a series of coated VACNT samples, where the thickness of the coating varies, we are also able to extract the resistance of the coated layer, *R*_s_, and its resistivity, *ρ*_coating_.

To carry out the electrochemical characterization, we use the setup sketched in Fig. 4b. The working electrode consists of either coated or uncoated VACNT samples, which are grown on Ni foil. The samples are partially taped with Kapton, leaving an effective area (*A*_electrode_) of approximately 0.8 cm × 0.8 cm exposed (see Fig. 1a). This working electrode (WE) is immersed in 1 M Na_2_SO_4_ aqueous electrolyte with Pt and Ag/AgCl as counter (CE) and reference electrodes (RE), respectively. We use an electrochemical workstation (CH Instruments, CHI 660E). All measurements are conducted at room temperature.

To demonstrate our approach, we perform electrochemical characterization on three VACNT samples, coated with different thickness layers of TiO_2_ (35, 50 and 75 cycles) using atomic layer deposition (ALD). The EIS data is acquired at open circuit potential with an oscillation amplitude of 5 mV and shown in Fig. 4c. Previous work has shown that this ALD-based, TiO_2_ coating bonds covalently onto the MWCNT exterior (*L* ≈ 100 μm over *d*_out_ ≈ 10 nm) and forms a side contact as shown schematically in Fig. 4a.^59,60^ In the first 20 ALD cycles, it is seen that the TiO_2_ coating grows at a slower rate than at a later stage, which likely comes from inhomogeneous nucleation and growth (Fig. S4†). Therefore, for the relative low numbers of ALD cycles here, we assume that we will not have complete coverage of the TiO_2_ coating, and include a leakage capacitance *C*_l_ (associated with ion adsorption directly onto and off of the CNTs) in the equivalent circuit model used to fit the EIS data (inset of Fig. 4c).

We note that this equivalent circuit model is similar to that of a ref. 61 for a suspended SWCNT coated with MnO_2_. We assume that the resistance of the CNT is negligible and that we do not observe strong ionic diffusion limitations within the electrolyte (Fig. S5†). In addition to the leakage capacitance *C*_l_, the other parameters in the equivalent circuit model include: the series resistance of the electrode and electrolyte, *R*_0_; the leakage resistance to the Ni foil substrate, *R*_l_; the electrical resistance of the TiO_2_, *R*_s_; the surface capacitance of the TiO_2_ coating *C*_s_ (associated with ion adsorption directly onto and off of the TiO_2_); the bulk faradaic contribution *Q* (with non-ideality factor *n* close to 1), and the Warburg diffusion resistance, *W* (associated with ionic diffusion into the TiO_2_); and finally, the key parameter of interest, the interfacial resistance between the CNT and TiO_2_ coating, *R*_i_.

Values for all fits are provided and trends with number of ALD layers are discussed in the ESI.† To further confirm that the values extracted from the equivalent circuit model fitting to EIS are sensible, we show in Fig. 4d that the sum of all the capacitances (*C*_l_, *C*_s_, and *Q*) extracted from the equivalent circuit model fitting to EIS are within 8% of the values of the capacitance *C* extracted from CV measurements (at 100 mV s^−1^ scanning rate) on the three coated VACNT samples within a 0–0.8 V voltage window.

To determine the 2D interfacial contact resistance *R*_2D_ from the interfacial resistance *R*_i_, we need to know how much of the CNT surface is coated (*A*_coat_):

10 *R*_2D_ = *R*_i_*A*_coat_

We use the CV scan to quantify the surface coverage of the coating *A*_coat_. The ratio of the leakage capacitance of the coated-VACNTs (extracted from the EIS measurements) to the capacitance of the uncoated-VACNTs (measured with CV to be *C*_0_ ≈ 2.47 mF) enables us to quantify the surface coverage of the coating *A*_coat_ using the expression:11
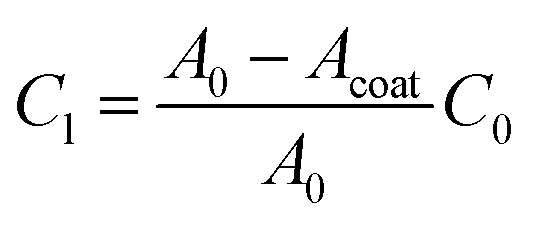
where the total side surface area (*A*_0_ ≈ 5.2 × 10^2^ cm^2^) is known from *n*_CNT_ ≈ 3 × 10^10^ cm^−2^ and the weight-gain method. The area of TiO_2_ coating (*A*_coat_) increases with the number of ALD cycles; however, even after 75 ALD cycles of ALD, surface coverage is only ∼82%. The normalized 2-dimensional contact resistance (*R*_2D_) can be defined by normalizing *R*_i_ with the total contact area (eqn (10)). We find a thickness independent *R*_2D_ of ∼85 Ω cm^2^ (Fig. 4e). Such a value is orders-of-magnitude greater than the typical contact resistance between metal (such as Ni) and graphene^62^ (similar to unfolded SWCNT surface) as 5 × 10^−6^ Ω cm^2^, which suggests a possible Schottky barrier at the interface between the CNT and the TiO_2_.

Additionally, we show that the resistivity of the coating, *ρ*_TiO_2__, can be obtained from *R*_s_, extracted from the EIS fitting. This value is important to know because of the type and quality (*i.e.*, crystal phase and morphology) of the coating grown on a high aspect ratio may differ from that grown using the same conditions on a flat 2D substrate. Indeed, here, we show from Raman spectroscopy, that the as-deposited TiO_2_ is a mixture of anatase and rutile phases (Fig. S4e†). We take the coated CNT to be analogous to a co-axial cable (eqn (12)), where the resistivity of the coating layer takes the form:12
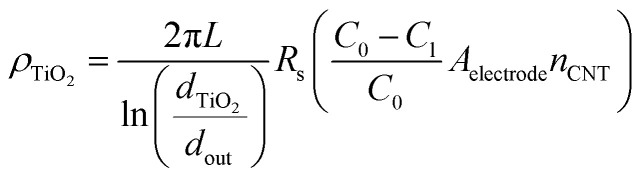
with the term in the latter bracket accounting for an effective number of coated CNTs. With the length (*L*) and diameter of TiO_2_ coated CNT (*d*_TiO_2__) obtained from SEM (Fig. S4†), and *n*_CNT_ determined *via* the weight-gain method, we find *ρ*_TiO_2__ from the slope of normalized *R*_s_*vs.* ln(*d*_TiO_2__/*d*_out_) plotted in Fig. 4e to be 2.6 × 10^10^ Ω cm. The value is of the same order of magnitude as an anatase TiO_2_ thin film calcined at 600 °C in air (10^10^ to 8 × 10^10^ Ω cm).^63^ In contrast a thin film with oxygen vacancies (*e.g.*, that has undergone hydrogen doping^64^ or thermal annealing in vacuum) is less resistive with 10^−2^ to 10^1^ Ω cm, independent of whether it is rutile or anatase phase.^65^ This hints that the TiO_2_ coating prepared by our ALD process likely has high crystallinity and few defects.

## 4. Conclusion

In order to systematically design lower resistance electrical and electrochemical devices using CNTs, we present approaches to measure the end contact resistance of CNTs in a VACNT array and the side contact resistances of ceramic coated CNTs in a VACNT array. These approaches can be performed on as-fabricated VACNT arrays or also on arrays that have been transplanted to different substrates. The approaches enable us to determine additional information such as the tunnel distance between the CNTs and the substrate as well as the quality of the coating (*i.e.*, its resistivity).

Our study highlights that contact resistances depend on the contact quality, which will be determined by the electronic structure of the substrate or coating material and its wettability with carbon (defining the tunneling barrier). While it is widely known that low-resistance end contacts are found between CNTs and metallic catalysts^17,32,34,35,51,66–69^ (*e.g.*, a bond as strong as 7.6 eV per bond could be formed between Co catalyst and SWCNT^70^), our results show that transplantation of CNTs (*e.g.*, on Ni at 400 °C) can still enable low resistively end contacts much below the typical CVD temperature (750 °C). These findings highlight that VACNT arrays can be transferred to substrates and devices on which direct CVD growth of the CNTs is not possible (*e.g.* flexible substrates, glass) such that their good electrical properties are maintained. However, even a substrate or coating that itself has high quality (*e.g.*, our ALD-coated TiO_2_ exhibits a low number of defects) may exhibit high contact resistance with the CNTs due, for example, to sub-optimal wetting.

## Conflicts of interest

There are no conflicts to declare.

## Supplementary Material

RA-009-C8RA10519G-s001
