# Supplementary material for: Characterization of contact resistances in ceramic-coated vertically aligned carbon nanotube arrays
Source: RSC Adv. 2019 Mar 4;9(13):7266–75. doi: 10.1039/c8ra10519g (PMC9087477; doi:10.1039/c8ra10519g)
Supplement: RA-009-C8RA10519G-s001 [file RA-009-C8RA10519G-s001.pdf]

Electronic Supplementary Material (ESI) for RSC Advances.
This journal is © The Royal Society of Chemistry 2019

Supplementary Information

Characterization of Contact Resistances in Ceramic-Coated Vertically Aligned Carbon Nanotube Arrays

Meng Li[a], Ning Yang[a], Vanessa Wood[*b], and Hyung Gyu Park[*a]

[a]*Department of Mechanical and Process Engineering, ETH Zürich, Zürich CH-8092, Switzerland. Email: parkh@ethz.ch*
[b]*Department of Information Technology and Electrical Engineering, ETH Zürich, Zürich CH-8092, Switzerland. Email: vwood@ethz.ch*

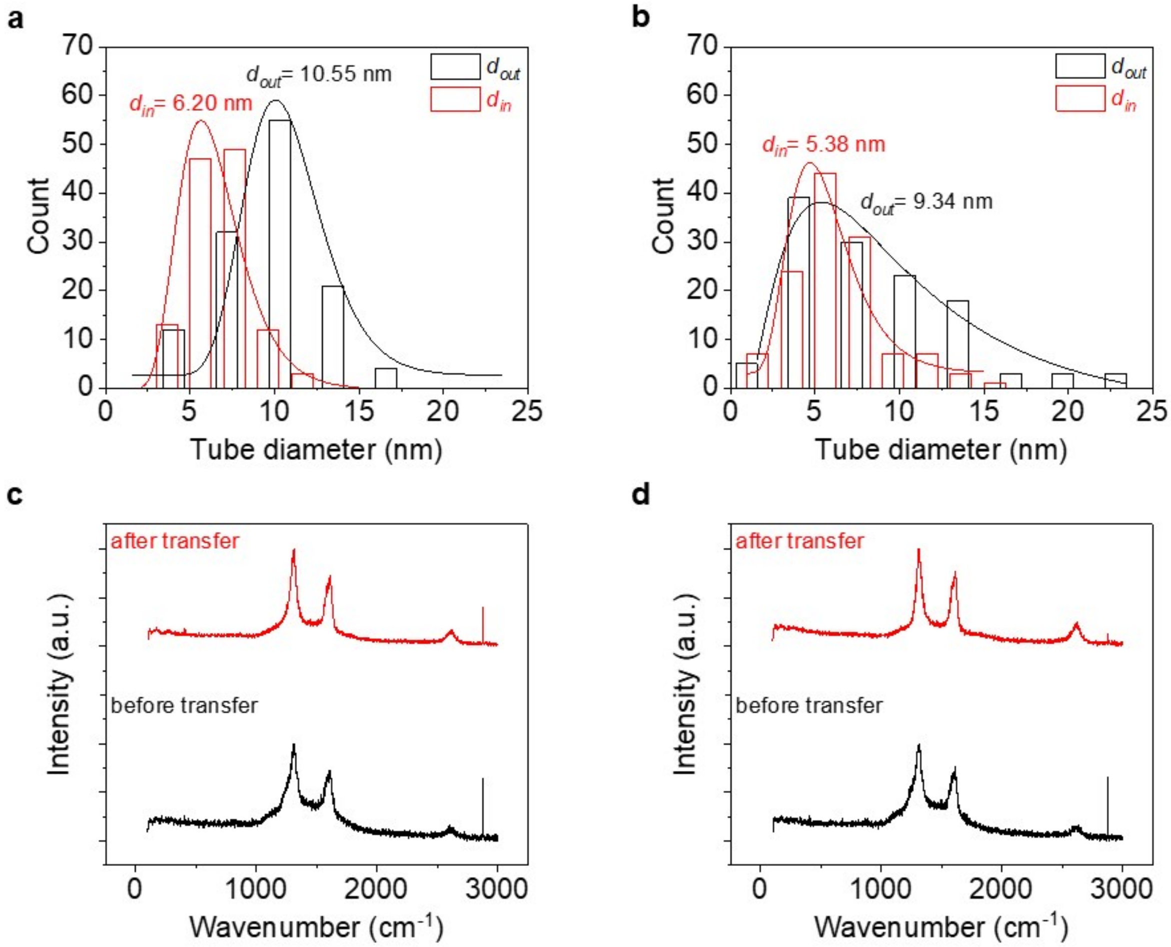

Fig. S1 Analysis of VACNTs diameters extracted from transmission electron microscopy (TEM) measurements for VACNT heights of (**a**) 17 µm, and (**b**) 43 µm. Fit assume a log-normal distribution. Raman spectra of VACNTs before and after transplantation for arrays of (**c**) 25 µm and (**d**) 38 µm in height.

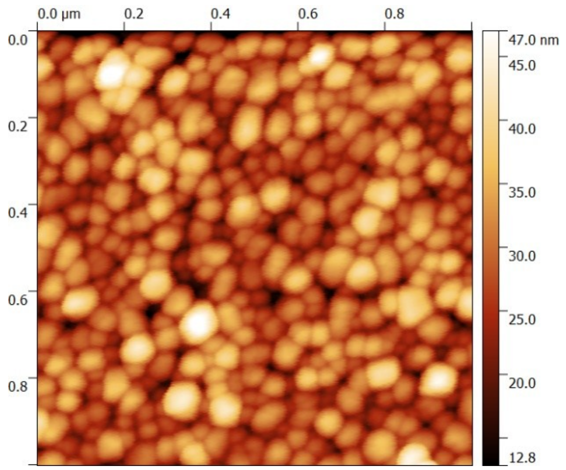

Fig. S2 AFM image of annealed catalysts on a Si substrate.

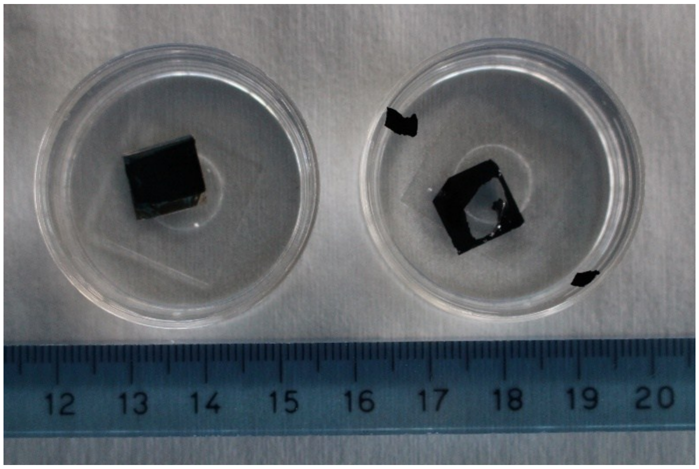

Fig. S3 Optical photo of as-transplanted VACNTs soaked in DI water: on a Ni foil (left) and on an FTO glass (right). VACNTs on Ni remain intact whereas they detach easily from FTO substrate.

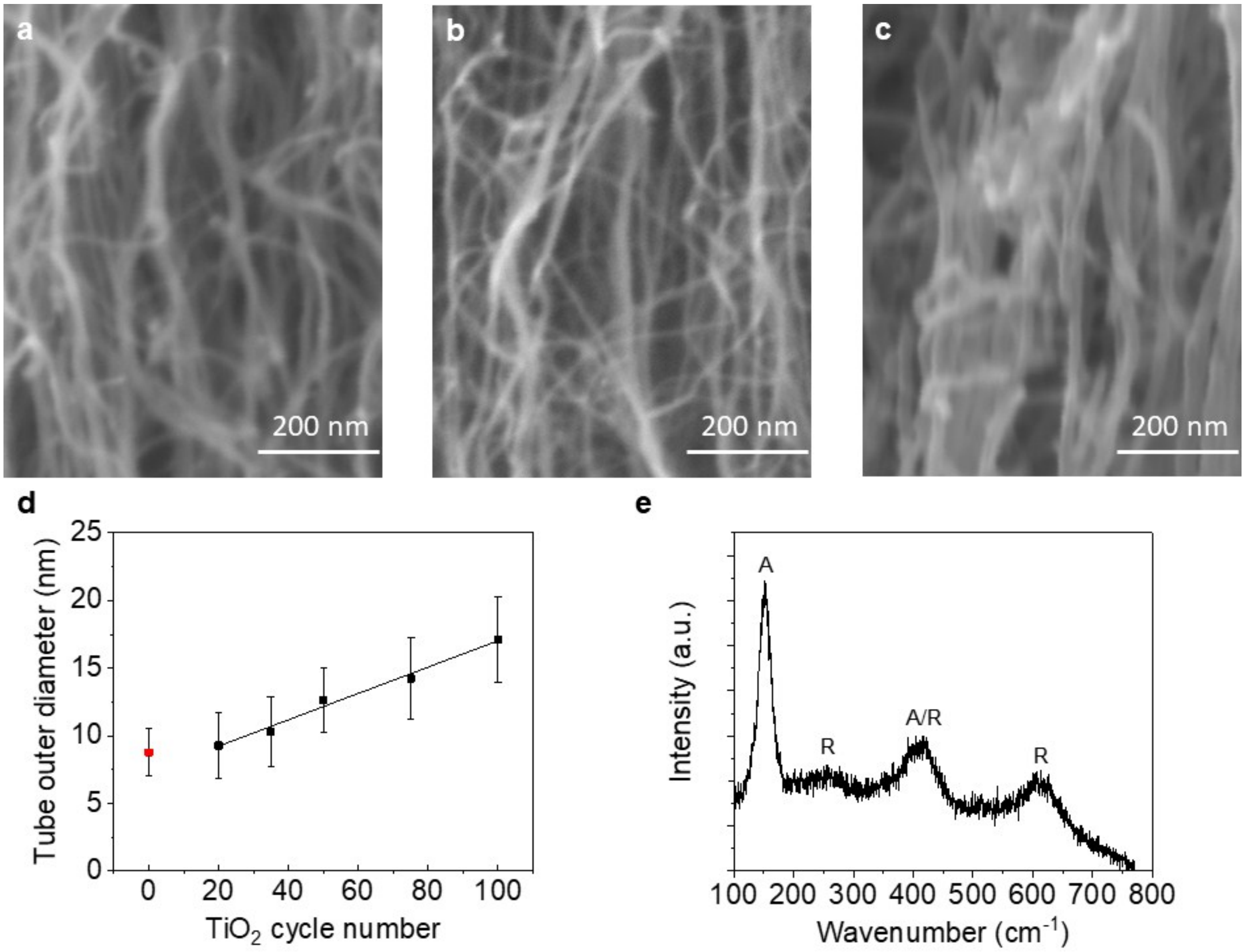

Fig. S4 Characterization of TiO_2 coating on VACNTs: High resolution SEM images of pristine VACNTs grown on Ni foil (**a**) near bottom-end, (**b**) near top-end, (**c**) VACNTs coated with 100 ALD cycles of TiO_2, (**d**) CNT outer diameter increase upon TiO_2 ALD and (**e**) Raman spectrum of TiO_2/VACNTs, where A and R represent anatase and rutile phase of TiO_2, respectively.

CVD growth on Ni foil (used for the electrochemical measurements) leads to more aligned CNTs and no apparent decay in number density, in contrast to growth on Si (used for transfer of the CNT arrays another substrate). In terms of the TiO_2 coating, the growth in the initial 20 ALD cycle is non-uniform and leads to an increase in CNT diameter at only ~0.05 nm/cycle, about half of the later stage. In our electrochemical analysis of coating, we therefore only consider more than 20 cycles.

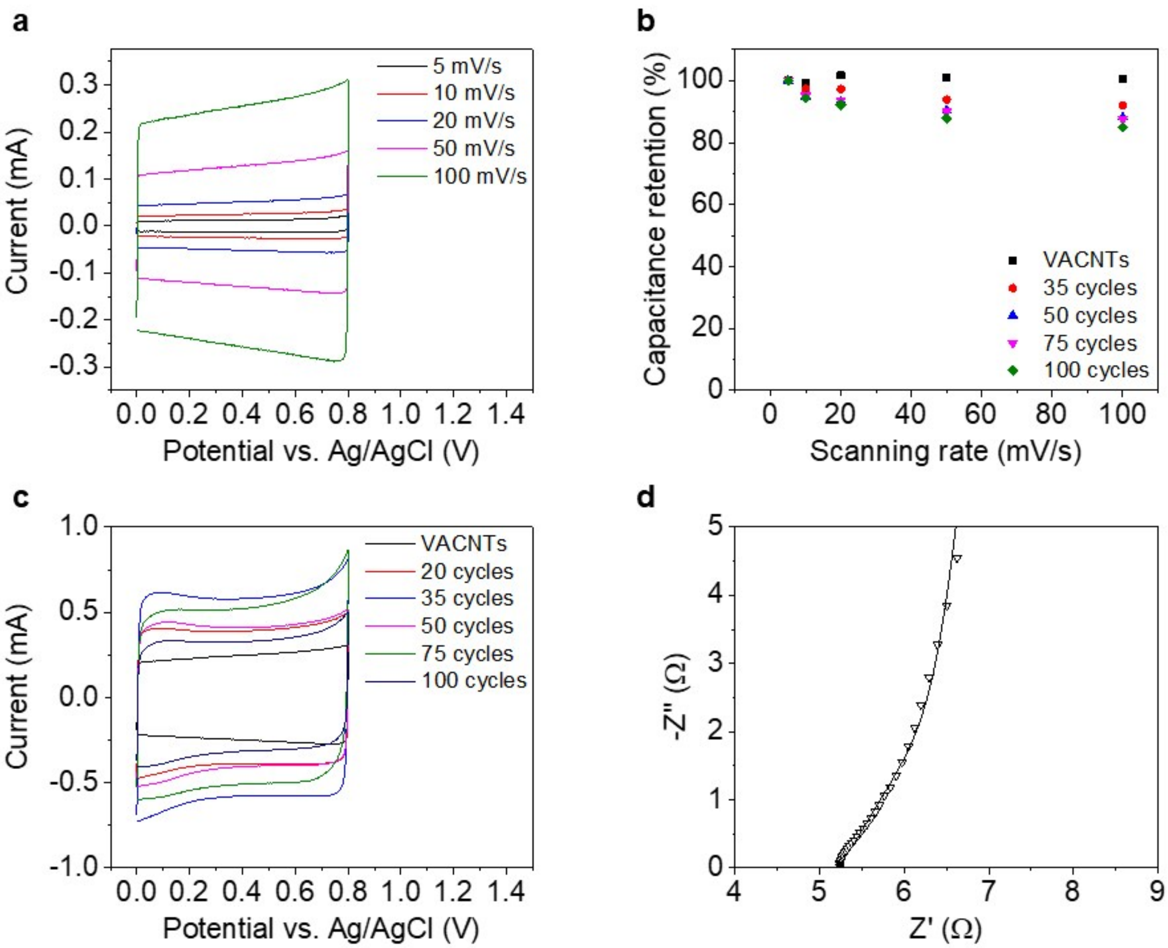

Fig. S5 Additional electrochemical characterizations of VACNTs and TiO_2/VACNTs: (**a**) Cyclic voltammetry of uncoated VACNTs at different scanning rates with (**b**) relative capacitance retention. The capacitance from CV scanning reveals no strong rate dependency up to 100 mV/s. With increasing number of ALD cycles, additional diffusion hindrance (but still less than 15%, even for 100 ALD cycles) is seen. We thus ignore effects such as ionic diffusion. (**c**) Cyclic voltammetry at 100 mV/s of TiO_2/VACNTs in different ALD cycles. (**d**) Nyquist plot of VACNTs coated with 100 ALD cycles of TiO_2, fitted using the model in **Fig. 4c**.

ALD cycle	R_0 (Ω)	R_l (Ω)	R_s (Ω)	R_i (Ω)	C_l (mF)	C_s (mF)	W ($\Omega \cdot s^{-0.5}$)	Q ($\Omega^{-1} \cdot s^n$)	n
35	4.97	31200	4.15	0.6	1.8	0.81	5.37	2.91×10^{-3}	0.947
50	5.29	26000	4.3	0.225	0.63	1.16	5.26	2.17×10^{-3}	0.94
75	5.47	18900	4.64	0.197	0.451	1.66	4.78	2.81×10^{-3}	0.942
100	5.2	36000	7.04	0.915	0.311	0.763	11.4	2.11×10^{-3}	0.943

Table S1 Summary of EIS fitting results from Fig. 4c.

Our model for EIS fitting is applicable up to 75 ALD cycles. After 100 ALD cycles, the coating begins to clog the channels in the VACNT forest, leading to diffusion limitations that are not captured with the simple equivalent circuit model.

Between 35 and 75 cycles the trends in the fitting parameters make sense. The series resistance R_0 increases with increasing cycle number, which may come from the increasing number of ALD reactions, which can slightly etch the CNTs, reducing their conductivity. The leakage resistance (R_l) and the leakage capacitance (C_l) both decrease while the capacitance at the surface between the TiO_2 coating and the electrolyte (C_s) increases with increasing number of coating cycles with is consistent with increase coverage of the CNTs. The resistance of the coating (R_s) increase with number of cycles indicating a progressively thicker coating. *W* and *Q* remain similar indicating that the properties of coating remain the same. A value of *n* close to 1 implies nearly ideal capacitive behavior from the electrode.
